# Interference of retroviral envelope with vaccine-induced CD8^+^ T cell responses is relieved by co-administration of cytokine-encoding vectors

**DOI:** 10.1186/s12977-017-0352-7

**Published:** 2017-04-27

**Authors:** Nadine Bongard, Dennis Lapuente, Sonja Windmann, Ulf Dittmer, Matthias Tenbusch, Wibke Bayer

**Affiliations:** 1 Institute for Virology, University Hospital Essen, University Duisburg-Essen, Virchowstr. 179, 45147 Essen, Germany; 2 Department of Molecular and Medical Virology, Institute of Hygiene and Microbiology, Ruhr-University Bochum, Bochum, Germany; 3 Institute of Clinical and Molecular Virology, University Hospital Erlangen, Friedrich-Alexander-University Erlangen-Nürnberg, Erlangen, Germany

**Keywords:** Retrovirus, Envelope, Vaccine, Friend virus, Friend retrovirus, Immunosuppression, Adjuvant, Immune modulation

## Abstract

**Background:**

Retroviral envelope (Env) proteins are known to exhibit immunosuppressive properties, which become apparent not only in retroviral infections, but also in gene-based immunizations using retroviral immunogens, where envelope interferes with the induction of CD8^+^ T cell responses towards another, simultaneously or subsequently delivered, immunogen.

**Results:**

In the Friend retrovirus mouse model, immunization with a plasmid encoding the Friend murine leukemia virus (F-MuLV) Leader-Gag protein resulted in induction of a strong GagL_85–93_-specific CD8^+^ T cell response, while the response was completely abrogated by co-immunization with an F-MuLV Env-encoding plasmid. In order to overcome this interference of retroviral envelope, we employed plasmids encoding the cytokines interleukin (IL) 1β, IL2, IL12, IL15, IL21, IL28A or granulocyte–macrophage colony-stimulating factor (GM-CSF) as genetic adjuvants. Co-application of plasmids encoding IL2, IL12, IL21, IL28A and especially GM-CSF rescued the induction of GagL_85–93_-specific CD8^+^ T cells in mice vaccinated with FV Leader-Gag and Env. Mice that were immunized with plasmids encoding Leader-Gag and Env and the cytokines IL1β, IL12, IL15, IL28A or GM-CSF, but not Leader-Gag and Env without any cytokine, showed significantly reduced viral loads upon a high-dose Friend virus challenge infection.

**Conclusions:**

Our data demonstrate the potency of cytokine-encoding vectors as adjuvants and immune modulators in composite vaccines for anti-retroviral immunization.

**Electronic supplementary material:**

The online version of this article (doi:10.1186/s12977-017-0352-7) contains supplementary material, which is available to authorized users.

## Background

The development of an effective anti-retrovirus vaccine has to overcome many challenges, among them the high sequence variability [1], the lack of immune correlates of protection [2, 3], and the immunosuppressive properties of some retroviral proteins. Numerous reports have shown an immunosuppressive effect exerted by retroviral envelope sequences, which is mostly attributed to an immunosuppressive domain located in the transmembrane envelope protein that is highly conserved in exogenous and endogenous retroviral envelope sequences and is an important feature of the ancient retrovirus envelope-derived mammalian syncytin proteins [4–7]. The immunosuppressive domain has recently been shown to evoke changes in chemokine and cytokine expression patterns, among them an induction of interleukin (IL) 6 and IL10 [8]. While the immunosuppressive effect of envelope in retroviral infection has been reported numerous times, an immunosuppressive effect has rarely been reported in the context of vaccine development. We have demonstrated before that a simultaneous or preceding immunization with an envelope-encoding vector will greatly diminish or even abrogate the CD8^+^ T cell induction by an otherwise effective vaccine against other immunogens such as another retrovirus-derived protein or the model antigen ovalbumin [9]. Interestingly, we detected envelope-specific IL10-producing CD4^+^ T cells in the immunized mice which may be involved in the suppression of CD8^+^ T cell responses towards other immunogens.

The immunosuppressive effect of retroviral envelopes is an important factor to consider in the development of an effective prophylactic vaccine, but it is also likely to affect vaccines used for therapeutic immunization in chronic retrovirus infections, as the immune system is envelope-experienced and furthermore continually exposed to the persistently expressed retroviral envelope protein, and could therefore interfere with therapeutic vaccination efforts. We demonstrated before that the envelope-mediated immune suppression can be circumvented by a sequential immunization where the CD8^+^ T cell inducing immunogen is applied as a first immunization separate from any envelope component. To generate a more generally applicable solution we now pursued the approach of a co-immunization with cytokine-encoding vectors as genetic adjuvants, aiming to change the cytokine milieu in a way that would favour the induction of CD8^+^ T cells. We evaluated the cytokines IL1β, IL2, IL12, IL15, IL21, IL28A and granulocyte–macrophage colony stimulating factor (GM-CSF) for their effect on CD8^+^ T cell induction in a DNA-based immunization, reasoning that these immunostimulatory cytokines might counter-act the envelope-mediated suppression either directly by supplying stimulatory signals for CD8^+^ T cells or indirectly through their activating effects on antigen-presenting cells.

The experiments presented here were performed in the murine Friend virus (FV) infection model. FV is an immunosuppressive retroviral complex [10] consisting of Friend murine leukemia virus (F-MuLV) and the replication-defective, pathogenic spleen focus forming virus, that causes severe splenomegaly and lethal erythroleukemia in adult mice of susceptible strains, while mice that are genetically resistant to the FV-induced disease develop a persistent infection without overt pathology [11]. Depending on the mouse strain, the FV infection can be a very stringent model for the development of prophylactic vaccines, or for therapeutic interventions such as immunotherapy or therapeutic immunization against an established, chronic infection [12–24]. While the FV infection of mice differs in target cell tropism and pathological mechanisms from human retrovirus infections, like the infection with human immunodeficiency virus or human T cell leukemia virus, it is assumed that basic immunological mechanisms that are responsible for the establishment of a chronic infection, and mechanisms required for virus control and clearance, are very similar for these retrovirus infections [25].

We demonstrate here that the co-immunization with F-MuLV envelope-encoding plasmid DNA impairs the induction of Leader-Gag specific CD8^+^ T cells, and show that co-immunization with plasmids encoding select cytokines relieves this suppression and confers strong protection to highly susceptible mice. To our knowledge, this is the first report showing that envelope-mediated immune suppression can be relieved by co-delivery of cytokines.

## Results

### Rescue of Env-suppressed GagL_85–93_-specific CD8^+^ T cell responses by co-immunization with cytokine-encoding plasmids

We reported before that the co-immunization with Leader-Gag and Env-encoding adenovirus-based vectors leads to an abrogation of the induction of CD8^+^ T cells specific for the Leader-Gag derived GagL_85–93_ epitope [9]. To test whether this suppression can be relieved through co-immunization with cytokine-encoding vectors, we used DNA-based immunizations, thereby excluding a possible influence of vector-derived epitopes or vector-specific immune responses, and combined plasmids encoding F-MuLV Leader-Gag and Env with plasmids encoding the murine cytokines IL1β, IL2, IL12, IL15, IL21, IL28A or GM-CSF; all these cytokines have important, direct or indirect, roles in CD8^+^ T cell priming and survival [26–29].

**Fig. 1:**
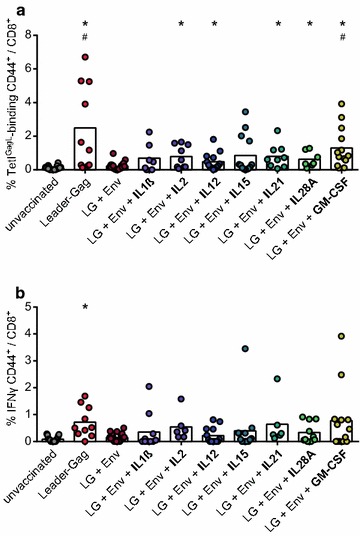
Induction of GagL_85–93_-specific CD8^+^ T cells by co-immunization with cytokine-encoding plasmids. CB6F1 mice were immunized once with a DNA plasmid encoding Leader-Gag, a mix of plasmids encoding Leader-Gag and Env (LG + Env), or a mix of plasmids encoding Leader-Gag, Env and cytokines as indicated; 25 µg of each plasmid were injected intramuscularly followed by in vivo electroporation. Two weeks after immunization, the frequency of GagL_85–93_-specific CD8^+^ T cells in blood cells was analysed by MHC I tetramer staining (**a**), the production of IFNγ by GagL_85–93_-specific CD8^+^ T cells was analysed by intracellular cytokine staining after peptide restimulation (**b**). Data were acquired in two to four independent experiments with three to four mice per group per experiment. *Each dot* indicates an individual mouse, the *bars* indicate the mean values. Data were analysed by Kruskall–Wallis One Way Analysis of Variance on Ranks and Dunn’s multiple comparison procedure, statistically significant differences (*P* < 0.05) compared to unvaccinated mice are indicated by *, significant differences compared to mice immunized with the combination of Leader-Gag and Env plasmids (LG + Env) are indicated by #

CB6F1 mice were immunized with DNA plasmids encoding Leader-Gag and Env (LG + Env) with and without cytokine-encoding plasmids, or as a control with Leader-Gag-encoding plasmid alone (see Additional file 1: Figure S1 for the vaccination scheme). When the frequency of GagL_85–93_-specific CD8^+^ T cells was analysed by MHC I tetramer staining 2 weeks after immunization, we found a strong induction of these cells in most mice immunized with the Leader-Gag-encoding plasmid alone, whereas the response was completely abrogated in mice immunized with the combination of Leader-Gag and Env plasmids (Fig. 1a). While co-immunization with the cytokines IL1β and IL15 resulted mostly in low frequencies of GagL_85–93_-specific CD8^+^ T cells which were not significantly different from the LG + Env vaccinated or the unvaccinated control groups, the co-immunization with IL2, IL12, IL21, IL28A and even more so with GM-CSF resulted in significantly improved induction of GagL_85–93_-specific CD8^+^ T cell responses. When we analysed the cytokine production of GagL_85-93_-specific CD8^+^ T cells, only mice that were immunized with the Leader-Gag plasmid alone had a significantly higher frequency of IFNγ-producing CD8^+^ T cells compared to unvaccinated mice (Fig. 1b); about half of the mice that were co-immunized with GM-CSF had levels of IFNγ-producing CD8^+^ T cells comparable to those induced by immunization with Leader-Gag plasmid alone, but responses did not reach statistically significant difference compared to mice immunized with LG + Env.

### Cytokine-adjuvanted immunization does not interfere with the induction of F-MuLV-binding antibodies

**Fig. 2:**
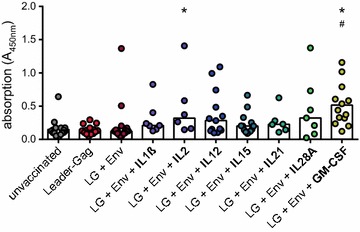
Binding antibody responses to plasmid combination vaccines. CB6F1 mice were immunized once with a DNA plasmid encoding Leader-Gag, a mix of plasmids encoding Leader-Gag and Env (LG + Env), or a mix of plasmids encoding Leader-Gag, Env and cytokines as indicated; 25 µg of each plasmid were injected intramuscularly followed by in vivo electroporation. Two weeks after immunization, blood samples were collected and F-MuLV-binding antibodies were analysed. The figure shows absorption values for plasma samples diluted 1:50 in PBS. Data were acquired in two to four independent experiments with three to four mice per group per experiment. *Each dot* indicates an individual mouse, *bars* indicate median values. Data were analysed by Kruskall–Wallis One Way Analysis of Variance on Ranks and Dunn’s multiple comparison procedure for statistical significance; statistically significant differences (*P* < 0.05) compared to unvaccinated mice are indicated by *, significant differences compared to mice immunized with the combination of Leader-Gag and Env plasmids (LG + Env) are indicated by #

The cytokines were selected for their potential effects on CD8^+^ T cell induction, but their modulatory effects might well introduce a new bias in the immune response and interfere with antibody induction; therefore, we analysed F-MuLV binding antibody levels after immunization. We found low antibody levels in mice immunized with Leader-Gag plasmid alone or with the combination of Leader-Gag and Env plasmids, and antibody levels of mice that were co-immunized with plasmids encoding IL1β, IL2, IL12, IL15, IL21 or IL28A were not significantly different; interestingly, mice that were co-immunized with GM-CSF had significantly higher binding antibody levels than both unvaccinated mice and mice vaccinated with LG + Env (Fig. 2). These findings demonstrate that the cytokine co-immunization, while rescuing the induction of GagL_85–93_-specific CD8^+^ T cells, does not interfere with antibody induction, and can even lead to improved antibody responses.

### Cytokine-adjuvanted immunization induces strong protection from high-dose FV challenge infection

**Fig. 3:**
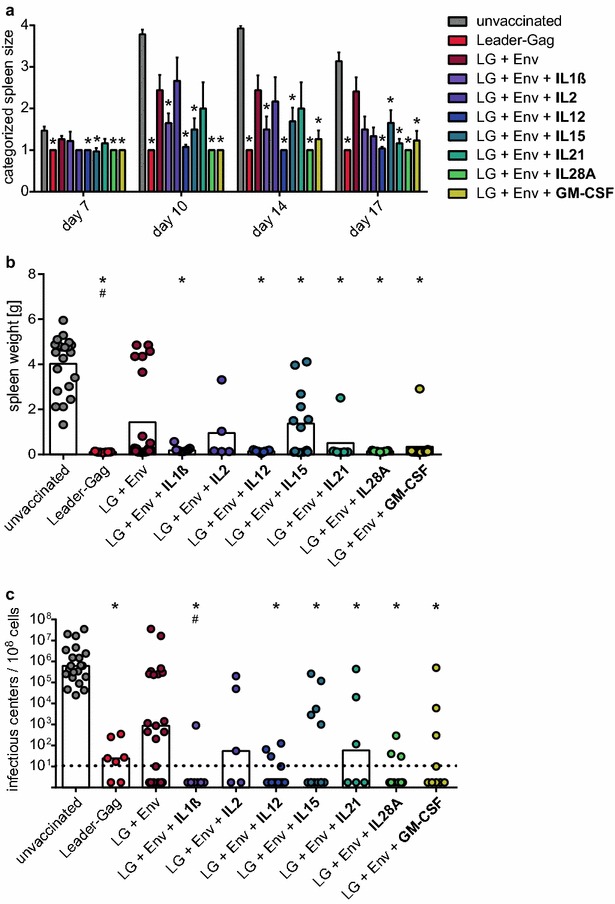
Protection from FV challenge infection by co-immunization with cytokine-encoding plasmids. CB6F1 mice were immunized once with a DNA plasmid encoding Leader-Gag, a mix of plasmids encoding Leader-Gag and Env (LG + Env), or a mix of plasmids encoding Leader-Gag, Env and cytokines as indicated; 25 µg of each plasmid were injected intramuscularly followed by in vivo electroporation. Three weeks after immunization, mice were infected with 5000 spleen focus forming units FV. The development of splenomegaly was monitored by palpation of the spleen twice a week (**a**). Three weeks after FV challenge, mice were sacrificed and spleen weight (**b**) and viral loads in spleen cells were determined (**c**). Data were acquired in two to four independent experiments with three to four mice per group per experiment. The *bars* in (**a**) indicate the mean values, whiskers indicate the standard error of the means. *Each dot* (**b**, **c**) indicates an individual mouse, bars indicate mean (**b**) or median (**c**) values. Data were analysed by Kruskall–Wallis One Way Analysis of Variance on Ranks and Dunn’s multiple comparison procedure, statistically significant differences (*P* < 0.05) compared to unvaccinated mice are indicated by *, significant differences compared to mice immunized with the combination of Leader-Gag and Env plasmids (LG + Env) are indicated by #

To determine if the rescued induction of GagL_85–93_-specific CD8^+^ T cells would result in adequate protection, we performed an FV challenge experiment and infected mice with the high dose of 5000 spleen focus forming units FV 3 weeks after the immunization. To monitor disease development, we palpated the spleens of mice twice a week (Fig. 3a) and found that while mice immunized with Leader-Gag-encoding plasmid alone showed no increase in spleen size over the entire observation period, mice that were immunized with plasmids encoding Leader-Gag and Env had severely enlarged spleens from day 10. Mice that were co-immunized with IL12, IL15, IL28A or GM-CSF on the other hand were able to control disease development and had significantly smaller spleens than unvaccinated mice over the entire observation period.

Three weeks after the FV challenge infection, we sacrificed the mice and removed the spleens. At this time point, unvaccinated mice had severely enlarged spleens, while spleens from mice that were immunized with Leader-Gag-encoding plasmid alone showed no increased weight (Fig. 3b). Spleens of about half of the mice that were immunized with Leader-Gag and Env plasmids had a severely increased weight, but mice that were co-immunized with IL1β, IL12, IL15, IL21, IL28A or GM-CSF were able to tightly control splenomegaly, and their spleens were significantly smaller than those of unvaccinated control mice. Similarly, when we analysed the viral load in spleen cells, unvaccinated mice all had very high viral loads, and while some mice immunized with LG + Env had low or even undetectable viral loads, about half of them had very high viral loads comparable with unvaccinated mice, and there was no statistically significant difference between LG + Env immunized and unvaccinated mice. On the other hand, mice that were immunized with Leader-Gag plasmid alone, or mice that were immunized with Leader-Gag and Env in combination with IL1β, IL12, IL15, IL21, IL28A or GM-CSF had significantly reduced viral loads that were below the detection limit in most of the mice (Fig. 3c).

To ascertain that the strong protection observed after co-immunization with cytokine-encoding plasmids was not mediated by direct effects of the cytokines on the FV infection, but in fact attributable to their effects on the FV-specific vaccine-induced immune response, we performed a control experiment and administered only the cytokine-encoding plasmids to CB6F1 mice. We used the cytokines IL1β, IL12, IL15, IL28A and GM-CSF as these cytokines had demonstrated the most profound effects on the viral loads in the co-immunization experiment. Three weeks after cytokine plasmid administration, mice were infected with 5000 spleen focus forming units FV. To monitor disease development, we palpated the spleens of infected mice twice a week, and did not find a significant difference between unvaccinated mice and mice that received the cytokine-encoding vectors (Additional file 2: Figure S2A); similarly, there was no significant difference between the groups in spleen weight (Additional file 2: Figure S2B) or spleen viral loads 3 weeks after FV infection (Additional file 2: Figure S2C). Some of the mice that received the IL12-encoding plasmid exhibited somewhat reduced spleen weight and spleen viral loads, which may indicate a direct or indirect effect of IL12 expression alone on FV infection. On the other hand, application of plasmids encoding IL1β, IL15, IL28A or GM-CSF had no effect on the course of the FV infection.

Our findings demonstrate that cytokine-adjuvanted immunization leads to a rescue of GagL_85–93_-specific CD8^+^ T cell responses from envelope-mediated immune suppression and induces strong protection from FV infection.

## Discussion

Retroviruses are notorious for their immunosuppressive potential, and while the severe immunodeficiency syndrome associated with progressive HIV infection has to be attributed to the depletion of CD4^+^ T cells in advanced infection, all retroviruses also exhibit acutely immunosuppressive properties. The immunosuppressive activity is generally assumed to be exerted by a domain in the transmembrane envelope protein which is highly conserved across exogenous and endogenous retroviral envelope sequences [5–8, 30]. The immunosuppressive properties of retroviral envelope are rarely addressed in immunization studies, and there are only few reports showing a dampening effect of envelope on the immune responses to other immunogens [31–34]. We have recently published the observation that immunization with an envelope-encoding adenovirus-based vector leads to a reduction or even abrogation of CD8^+^ T cell induction to other simultaneously or subsequently delivered immunogens [9]. In contrast to other reports [31], our experiments did not demonstrate a relief of the suppression when the envelope vaccine and the CD8^+^ T cell inducing vaccine were spatially separated, and while we did not find a significantly changed induction of regulatory CD4^+^ T cells by envelope-encoding adenovirus in comparison to adenovirus encoding another transgene, we did find production of IL10 by envelope-specific CD4^+^ T cells, hinting at the establishment of a suppressive cytokine milieu. Because of these changes in the cytokine milieu, we addressed the suppressive effect in the context of DNA immunization and sought to relieve suppression by co-immunization with cytokine-encoding plasmids as genetic adjuvants. For this purpose, we selected the pro-inflammatory cytokines IL1β, IL2, IL12, IL15, IL21, IL28A and GM-CSF, all of which can directly or indirectly influence the induction or maintenance of T cell responses [26–29].

We found that IL1β and IL15 had only moderate effects on the induction of GagL_85–93_-specific CD8^+^ T cells, whereas IL2, IL12, IL21 and IL28A led to a rescue of GagL_85–93_-specific CD8^+^ T cell induction in most of the co-immunized mice; but clearly, GM-CSF had the most profound effect on induction of GagL_85–93_-specific CD8^+^ T cell responses in the presence of envelope. GM-CSF has been described before as an adjuvant in DNA- or adenovirus-based immunization against HIV infection or against model antigens [35–38], in virus-like particle-based immunization against Hanta virus infection [39], and in experimental cancer immunization in pre-clinical [40] and clinical studies [41, 42]. In all of these studies, GM-CSF was reported to have an enhancing effect on CD8^+^ T cell responses, which it may exert indirectly by promoting the activation and maturation of dendritic cells [43].

It is assumed that the development of a highly potent anti-retroviral vaccine requires the induction of both cellular and humoral immune responses [44–46]; therefore employing a CD8^+^ T cell promoting cytokine to relieve the envelope-mediated suppression should not interfere with the induction of antibodies and thereby introduce a new bias. Importantly, none of the cytokines tested in our experiments had a diminishing effect on antibody induction, and GM-CSF co-immunization actually resulted in an increase in binding antibody levels, which is well in accordance with the literature that reports a simultaneous promotion of CD8^+^ T cell and antibody responses by GM-CSF [36]. We showed recently that a rationally designed adenovirus-based vaccine could confer a high level of protection from FV challenge infection similar to the gold standard, attenuated anti-FV vaccine F-MuLV-N, albeit by different mechanisms [9]; indeed the DNA-based, cytokine-adjuvanted vaccine that we present here proves similarly effective, and furthermore might be applicable as a therapeutic vaccine as induction of CD8^+^ T cell responses does not rely on the vaccinee being envelope naïve.

The envelope-mediated immunosuppression is not only of concern for the development of prophylactic anti-retroviral vaccination strategies, but also for therapeutic vaccine approaches. A chronically infected individual is envelope-experienced and chronically envelope-exposed; furthermore, the chronic phase of FV infection is characterized by an expanded population of regulatory T cells (Treg) [47–51] that are creating an immunosuppressed state and interfering with effector functions of CD8^+^ T cells. Immunotherapeutic interventions targeting regulatory T cells, such as depletion of Treg cells or blocking of inhibitory ligands, have shown effective in reducing viral loads in the chronic FV infection [22, 23]; a combination of a therapeutic immunization using cytokine-adjuvanted DNA vaccines, which circumvents the suppression by envelope, with immunotherapeutic interventions such as Treg depletion and the blockade of inhibitory ligands may result in additive or even synergistic effects on the chronic FV loads and shall be investigated in the future. While IL21 was not the most effective cytokine in the prophylactic immunization, it may be a promising candidate for therapeutic application. IL21 can act on many cellular targets, among them antigen presenting cells such as dendritic cells, but also directly on T cells [52], and it was suggested to be able to substitute for IL2 as a T cell growth factor [53].

Our results show that select cytokines are able to relieve the immunosuppression exerted by retroviral envelope and suggest that cytokine-adjuvanted vaccines may be powerful tools in anti-retroviral vaccine design.

## Methods

### Cells and cell culture

A murine fibroblast cell line from Mus dunni [54] was maintained in RPMI medium (Invitrogen/Gibco, Karlsruhe, Germany) supplemented with 10% heat-inactivated fetal bovine serum (Invitrogen/Gibco) and 50 µg/ml gentamicin; cells were maintained in a humidified 5% CO_2_ atmosphere at 37 °C.

### Plasmid DNA

The plasmids encoding the F-MuLV proteins Env or Leader-Gag under control of the cytomegalovirus immediate early promoter have been described before [55]. Plasmids encoding the murine cytokines IL2, IL12, IL15, IL21 or GM-CSF are based on the pShuttle-CMV expression plasmid and have been described before [16]. Plasmids encoding murine IL1β or IL28A were constructed by PCR amplification of the coding sequences from cDNA of an influenza-infected mouse lung and subcloning into the pVax plasmid (ThermoFisher Scientific, Darmstadt, Germany). Since mature IL1β does not have a conventional export signal, the leader peptide of the tissue plasminogen activator was added at the N-terminus to allow efficient secretion of the cytokine.

All plasmids were purified by cesium chloride gradient centrifugation and absence of endotoxin contamination was verified by Limulus amebocyte lysate assay (detection limit 0.1 EU/ml; ThermoFisher Scientific, Schwerte, Germany).

### Mice

Female CB6F1 hybrid mice (BALB/c x C57BL/6 F1; H-2^b/d^ Fv1^b/b^ Fv2^r/s^ Rfv3^r/s^) and female BALB/c mice were purchased from Charles River Laboratories (Sulzfeld, Germany). All mice were used when they were between 8 and 9 weeks of age.

### Immunization

CB6F1 mice were immunized intramuscularly into the M. gastrocnemius with 25 µg each of the respective plasmids in 30 µl PBS. Immediately following the DNA injection, in vivo electroporation was performed using the BTX AgilePulse system (BTX Molecular Delivery Systems, Holliston, MA) or the Ichor electroporation system (Ichor Medical Systems, San Diego, CA) using electroporation protocols described before [56, 57]. Electroporation with both devices gave similar results.

### FV and challenge infection

Uncloned, lactate dehydrogenase-elevating virus (LDV)-free FV stock was obtained from BALB/c mouse spleen cell homogenate (10%, wt/vol) 14 days post infection with a B cell-tropic, polycythemia-inducing FV complex [58]. CB6F1 mice were challenged by the intravenous injection of 5000 spleen focus-forming units.

The development of FV-induced disease was monitored by palpation of the spleens of infected mice twice a week under general anaesthesia, and spleen sizes were rated on a scale ranging from 1 (normal spleen size) to 4 (severe splenomegaly) as described previously [59]. If mice showed overt signs of severe disease before the end of the experiment as rated by pre-determined termination criteria, they were euthanized and excluded from further analysis.

### Infectious center assay

Mice were sacrificed 21 days after FV infection by cervical dislocation, the spleens were removed and weighed, and single-cell suspensions were prepared. Serial dilutions of isolated cells were seeded onto *M. dunni* cells, and cells were incubated under standard tissue culture conditions for 3 days. When cells reached ~100% confluence, they were fixed with ethanol, labelled with F-MuLV Env-specific Mab 720 [60], and then with a horseradish peroxidase-conjugated rabbit anti-mouse Ig antibody (Dako, Hamburg, Germany). The assay was developed using aminoethylcarbazole (Sigma-Aldrich, Deisenhofen, Germany) as substrate to detect foci. Resulting foci were counted, and infectious centers (IC)/10^8^ cells were calculated.

### Binding antibody ELISA

For the analysis of F-MuLV-binding antibodies, MaxiSorp ELISA plates (Nunc, Roskilde, Denmark) were coated with whole F-MuLV antigen (5 µg/ml). After coating, plates were blocked with 10% fetal calf serum in PBS, and incubated with plasma dilutions. Binding antibodies were detected using a polyclonal rabbit-anti-mouse HRP-coupled anti-IgG antibody and the substrate tetramethylbenzidine (TMB+; both Dako Deutschland GmbH, Hamburg, Germany); absorption at 450 nm wavelength was analysed after addition of an equal volume of 1 N H_2_SO_4_.

### Tetramer staining of F-MuLV-specific CD8^+^ T cells

F-MuLV-specific CD8^+^ T cells were analysed in peripheral blood 2 weeks after immunization. Erythrocytes were lysed, and cells were stained with PE-coupled MHC I tetramer (containing the H-2D^b^ restricted F-MuLV gag-leader epitope AbuAbuLAbuLTVFL in which cysteine residues of the original amino acid sequence GagL_85–93_ (CCLCLTVFL) were replaced by amino-butyric acid (Abu) to prevent disulfide bonding [61]; MBL, Woburn, MA), PerCP-anti-CD43, eFluor450-anti-CD8, BV510-anti-CD44 (Becton–Dickinson, Heidelberg, Germany) and Fixable Viability Dye eFluor 780 (eBioscience, Frankfurt, Germany).

Data were acquired on an LSR II flow cytometer (Becton–Dickinson, Mountanview, CA) and analyzed using FlowJo software (Tree Star, Ashton, OR).

### Intracellular cytokine staining

For the analysis of effector molecules of GagL_85–93_-specific CD8^+^ T cells, cells were stimulated for 6 h in vitro with 1 µg/ml Abu-modified GagL_85-93_ peptide (AbuAbuLAbuLTVFL; Abu-modified from the original sequence CCLCLTVFL) in the presence of 2 µg/ml brefeldin A. Cells were stained with eFluor450-anti-CD8, PerCP-anti-CD43, BV510-anti-CD44, and FITC-anti-interferon γ (IFNγ; Becton–Dickinson, Heidelberg, Germany).

Data were acquired on an LSR II flow cytometer (Becton–Dickinson, Mountanview, CA) and analysed using FlowJo software (Tree Star, Ashton, OR).

### Statistical analyses

Statistical analyses were performed using the software SigmaStat 3.1 (Systat Software GmbH, Erkrath, Germany), testing with Kruskal–Wallis One-Way Analysis of Variance on Ranks and Dunn’s multiple comparison procedure.

## Additional files

**Additional file 1: Figure S1.** Experimental layout. CB6F1 mice were immunized with 25 µg plasmid DNA each encoding Leader-Gag, Leader-Gag and Env, or Leader-Gag, Env and a cytokine. Antibody and CD8^+^ T cell responses were analysed two weeks after immunization. Three weeks after immunization, mice were challenged with 5000 SFFU FV, and plasma and spleen viral loads were analysed ten days and 21 days after FV infection, respectively.

**Additional file 2: Figure S2.** Immunization with cytokine-encoding vectors alone. CB6F1 mice were immunized once with DNA plasmids encoding cytokines as indicated; 25 µg of plasmid were injected intramuscularly followed by in vivo electroporation. Three weeks after immunization, mice were infected with 5000 spleen focus forming units FV. The development of splenomegaly was monitored by palpation of the spleen twice a week (A). Three weeks after FV challenge, mice were sacrificed and spleen weight (B) and viral loads in spleen cells were determined (C). Data were acquired in one experiment. The bars in (A) indicate the mean values, whiskers indicate the standard error of the means. Each dot (B, C) indicates an individual mouse, bars indicate mean (B) or median (C) values. The grey-bordered dots above the grey lines (B, C) indicate mice that were sacrificed before the end of the experiment due to severe disease development; they were not included in data analysis. Data were analysed by Kruskall-Wallis One Way Analysis of Variance on Ranks and Dunn’s multiple comparison procedure; n.s. = not significant compared to unvaccinated mice (*P* > 0.05).
